# Use of NanoSIMS to Identify the Lower Limits of Metabolic Activity and Growth by *Serratia liquefaciens* Exposed to Sub-Zero Temperatures

**DOI:** 10.3390/life11050459

**Published:** 2021-05-20

**Authors:** Petra Schwendner, Ann N. Nguyen, Andrew C. Schuerger

**Affiliations:** 1 Space Life Sciences Lab, Department of Plant Pathology, University of Florida, 505 Odyssey Way, Exploration Park, Merritt Island, FL 32953, USA; schuerg@ufl.edu; 2 Jacobs, NASA Johnson Space Center, Houston, TX 77058, USA; lan-anh.n.nguyen@nasa.gov

**Keywords:** Mars astrobiology, psychrotolerant bacteria, NanoSIMS, isotopes, hypopiezotolerant bacteria

## Abstract

*Serratia liquefaciens* is a cold-adapted facultative anaerobic astrobiology model organism with the ability to grow at a Martian atmospheric pressure of 7 hPa. Currently there is a lack of data on its limits of growth and metabolic activity at sub-zero temperatures found in potential habitable regions on Mars. Growth curves and nano-scale secondary ion mass spectrometry (NanoSIMS) were used to characterize the growth and metabolic threshold for *S. liquefaciens* ATCC 27,592 grown at and below 0 °C. Cells were incubated in Spizizen medium containing three stable isotopes substituting their unlabeled counterparts; i.e., ^13^C-glucose, (^15^NH_4_)_2_SO_4_, and H_2_^18^O; at 0, −1.5, −3, −5, −10, or −15 °C. The isotopic ratios of ^13^C/^12^C, ^15^N/^14^N, and ^18^O/^16^O and their corresponding fractions were determined for 240 cells. NanoSIMS results revealed that with decreasing temperature the cellular amounts of labeled ions decreased indicating slower metabolic rates for isotope uptake and incorporation. Metabolism was significantly reduced at −1.5 and −3 °C, almost halted at −5 °C, and shut-down completely at or below −10 °C. While growth was observed at 0 °C after 5 days, samples incubated at −1.5 and −3 °C exhibited significantly slower growth rates until growth was detected at 70 days. In contrast, cell densities decreased by at least half an order of magnitude over 70 days in cultures incubated at ≤ −5 °C. Results suggest that *S. liquefaciens*, if transported to Mars, might be able to metabolize and grow in shallow sub-surface niches at temperatures above −5 °C and might survive—but not grow—at temperatures below −5 °C.

## 1. Introduction

The understanding of microbial–niche interactions, the geological and thermodynamical contexts of frozen habitats, the microbiome, and the geochemical physical state of habitable niches are fundamental to describing the potential for the presence of microorganisms, their activity, detritus, and biosignatures on both Earth and in extraterrestrial environments. On Earth, microbial communities have been found in frozen habitats such as glaciers, permafrost, and in ice at both polar regions where liquid water is only available intermittently, seasonally, or in insulated microenvironments [1,2,3,4,5,6]. Impurities in the ice can depress the freezing point of water and create thin networks of unfrozen water in icy environments which form microhabitats for microorganisms to survive and thrive [7]. However, these niches are characterized by low thermal energy, limited availability of nutrients, low water availability and water activity, and slower diffusion of metabolic waste products which can lead to increased toxicity. High solute concentrations cause osmotic imbalance posing a challenge for microbial survival and growth [8,9,10,11]. 

For Mars, different icy terrains have been described such as polar caps, lake deposits, and ground ice; condensation on spacecraft struts; and permafrost. For Europa, an ice shell of 15 to 25 km thickness has been described above a liquid ocean in contact with a metal core likely possessing oceanic hydrothermal features [12,13]. Ice-dominated locations have been suggested as possible refugia of habitable niches that may support biological growth or may contain biosignatures of past or present life [14,15,16,17,18]. Consequently, environmental conditions in cold and/or frozen niches on other planetary bodies should be studied to determine whether they can provide potential habitats for microbial life to metabolize and grow in general, and for potential astrobiology model organisms such as *Serratia liquefaciens,* in particular. 

*Serratia liquefaciens* is a cold-adapted, facultative anaerobic, Gram-negative, motile bacterium that has been demonstrated to be able to grow at simulated Martian conditions of low pressure (7 hPa), low temperature (0 °C), and a CO_2_-enriched anoxic atmosphere [19,20,21]; henceforth called low-PTA conditions. Furthermore, *Serratia* spp. have been isolated from spacecraft assembly facilities and have been recovered from samples taken onboard the International Space Station and Russian spacecraft [22]. Their presence in spacecraft-associated environments makes *Serratia* spp. plausible candidates to be transferred to extraterrestrial bodies. *Serratia liquefaciens* has been described as an ecological generalist under optimal growth conditions found in soil, water, plants, and digestive tracts of humans and of various animals. Among the various carbon sources that can be metabolized are for example carbohydrates such as glucose, alcohols, carboxylic acids, amino acids, and aromatic or brominated substrates. However, the incubation at simulated Martian low-PTA conditions led to reductions in potential substrates that can be used for growth [21], suggesting changes in metabolic activity and growth rates.

The most likely candidates to investigate for growth and metabolic potential at subzero temperatures would be psychrophiles. Psychrophiles are microorganisms that colonize a diverse range of cold environments, grow optimally below +10 °C, and require cold conditions to thrive. Potential record holders for growth and metabolic activity in sub-zero temperatures have been investigated (*Psychrobacter arcticus* down to −15 °C [23]). For two isolates from an Antarctic glacier *Paenisporosarcina* sp. B5 and *Chryseobacterium* sp. V3519-10, metabolic activity such as DNA repair, protein synthesis, and respiration—but not cell division—has been confirmed down to −33 °C [24]. The current lower limit for metabolic activity in microorganisms is −39 °C [25,26]. However, the likelihood of the presence of viable psychrophiles in spacecraft assembly cleanrooms is very low since spacecraft are assembled in strictly controlled clean rooms where microbial contamination is very limited and the temperature is set at moderate temperatures around 21 °C, with a maximum of 27 °C. 

The majority of potential cultivable microbial hitchhikers on a Mars-bound rover or lander are mesophilic bacteria and a few cold-adapted bacteria [27,28,29]. While psychrophiles are optimally adapted to low temperatures, cold-adapted bacteria which remain active between 0 and 40 °C and mesophilic bacteria growing at moderate temperatures of 20–45 °C struggle at temperatures below their optimal growth range and face several challenges that lead to various indirect and direct physiological changes. Examples are: a loss of membrane fluidity, proteins including membrane-bound enzymes may denature and lose flexibility, consequently affecting enzyme activity and nutrient transport [30]. Replication, transcription, and translation are inhibited due to changes in the secondary structures of DNA and RNA [31]. Among the direct effects are the alteration of cell composition, differential nutrient requirements, lower enzyme activities, and the potential for intracellular ice crystal formation which can lead to mechanical damage and a decreased growth rate [32]. 

Since the microbial biomass turnaround in extreme environments is slow, commonly applied techniques to measure growth, biomass, and metabolism can reach their limits due to the measurement thresholds, detection limits, and increased probability of introducing experimental bias. The development of stable-isotope methods has been revolutionary for identifying metabolic activity in extreme environments. 

Nano-scale secondary ion mass spectrometry (NanoSIMS) uses a high spatial resolution ion probe to create nanoscale isotopic maps of cells, allowing identification, quantification, and visualization of the incorporation of labeled substrates within single organisms [33]. Consequently, the metabolic states of cells can be probed when the growth media are doped with stable isotopes based on the abundance of the isotopes in cells [34,35]. 

We performed a series of experiments to determine the boundaries of metabolism and growth for the bacterium, *Serratia liquefaciens*, at freezing and sub-zero temperatures by supplementing the growth medium with three isotopically labeled substrates including ^13^C-glucose, (^15^NH_4_)_2_SO_4_, and H_2_^18^O. The uptake and incorporation into cell biomass were assessed using NanoSIMS by simultaneously measuring the ^13^C/^12^C, ^12^C^15^N/^12^C^14^N, and ^18^O/^16^O ratios in the cells. The astrobiological model organism, *S. liquefaciens*, is a cold-adapted bacterium with an optimal growth rate at 30 °C that was selected for these experiments because it can metabolize and grow on single carbon sources and under simulated Martian low-PTA conditions. The goal of the research was to characterize the lower limits of growth and metabolism at sub-zero temperatures with *S. liquefaciens* as a prelude for conducting more complex experiments under simulated Martian low-PTA conditions. This is the first step towards assessing the potential of *S. liquefaciens* to be able to survive, metabolize, and grow in sub-zero niches that have been suggested as potential habitats for extinct or extant life on Mars. 

## 2. Materials and Methods

### 2.1. Microbial Enrichment Protocols

Cultures of *S. liquefaciens* ATCC 27,592 were maintained on tryptic soy agar (TSA; Becton, Dickinson and Company, Sparks, MD, USA), and plates incubated for 24 h at 30 °C. Liquid cultures in Spizizen medium [36] were created using bacterial colonies from TSA. Spizizen medium was composed of 470 mL of a 1× Spizizen salts solution, 5 mL of an iron-sulfate solution (1.92 g/L of Fe_2_(SO_4_) + 3.58 g/L of diethylenetriaminepentaacetic acid; DTPA), 2.5 g of NaCl, and 25 mL of a micronutrient solution. The micronutrient stock solution was prepared according to the following recipe: MnSO_4_ · H_2_O (0.246 g/L), ZnSO_4_ · H_2_O (0.264 g/L), H_3_BO_3_ (0.576 g/L), CuSO_4_ · 5 H_2_O (0.152 g/L), and molybdenum (0.0074 μM (NH_4_)_6_Mo_7_O_24_). Either 10 mM or 20 mM glucose was added as the sole carbon source. The medium was filter-sterilized through 0.2 µm filters (polyethersulfone membrane, Fisher Scientific, Waltham, NA, USA) to prevent the precipitation of the salts that occurred during autoclaving. 

One hundred and eighty microliters of Spizizen medium were dispensed into each well of a 96-well plate. Each well was inoculated with 20 µL of fresh liquid culture of *S. liquefaciens* in Spizizen medium. The starting concentration of the inoculum was approximately 2 × 10^7^ cells/mL. Cells were incubated at 0, −1.5, −3, −5, −10, and −15 °C for 70 days to determine survivability and growth (i.e., derive a growth curve for specific temperatures and glucose concentrations). At 7-day intervals, cell concentrations were estimated by applying most Probable Number (MPN) assays [20,21]. The medium remained liquid at temperatures down to −5 °C but froze at −10 and −15 °C.

### 2.2. Cell Enumeration via MPN

Growth rates in unlabeled Spizizen media were determined to estimate cell densities per milliliter for specific temperatures and either 10 or 20 mM glucose. In brief, 1-mL samples (i.e., 200 µL aliquots from five individual wells were bulked) of the incubated cells were 10-fold serially diluted per treatment. Twenty microliters per well of each dilution were pipetted into two columns each of a 96-well microtiter plate filled with 180 µL of trypticase soy broth (TSB; Becton, Dickinson and Company, Sparks, MD, USA). MPN plates were incubated at 30 °C. After 24 h, the numbers of positive (i.e., turbid) wells were determined and the viable cell concentrations per milliliter calculated. 

### 2.3. Isotope Labeling Experiments 

Subsequently, cells of *S. liquefaciens* were grown in Spizizen medium containing three stable isotopes substituting their unlabeled counterparts: 20 mM ^13^C-glucose (^13^C, 99%), (^15^NH_4_)_2_SO_4_ (^15^N, 99%), and H_2_^18^O (^18^O, 97%). All labeled chemicals were ordered from Cambridge Isotope Laboratories, Inc. (Tewksbury MA, USA). Twenty microliters of cell suspensions were added to each well previously filled with 180 µL labeled Spizizen medium. The microtitre plates were incubated at 0, −1.5, −3, −5, −10, or −15 °C for 35 days for NanoSIMS imaging of cells and 70 days for determining growth rates via MPN assays, as described above. In addition to these samples, two controls were added that included: (1) UV-killed *S. liquefaciens* cells were incubated in labeled Spizizen medium at 0 °C for 35 days; and (2) live *S. liquefaciens* cells were incubated in unlabeled Spizizen medium at 0 °C for 35 days.

### 2.4. Scanning Electron Microscopy (SEM)

For subsequent SEM and NanoSIMS analyses, cells were harvested after 35 days of incubation by withdrawing 200-µL aliquots from five randomly selected wells (i.e., a total of 1 mL), pelleted via centrifugation, and fixed in 2% glutaraldehyde in 1× phosphate buffered saline (PBS). After 30 min of fixation at room temperature (~23 °C), the cell suspensions were spread onto Isopore HTTP 0.2-µm filters (Merck Millipore Ltd., Tullagreen, Ireland) that were previously treated with 0.01% poly-L-lysine solution and placed onto 2% water agar plates. After the initial glutaraldehyde solutions were drawn through the filters by the water agar, filters were placed in 2% glutaraldehyde (2 h at 23 °C), washed three times with 1× PBS, post-fixed in 1% PBS-buffered osmium tetroxide, washed thrice with PBS, and thrice with filter-sterilized deionized water (SDIW). Cell preparations were then dehydrated through an ethanol (EtOH) series at 10, 25, 50, 75, 90 and 100% EtOH. The samples were critical-point dried (CPD) using a Tousimus CPD system at the Interdisciplinary Center for Biotechnology Research (ICBR) at the University of Florida (UF). The samples were sputter coated with gold-palladium and imaged with a Hitachi SU5000 SEM at the ICBR to map the samples prior to analyzing the cells in the NanoSIMS (Supplemental Figure S1).

### 2.5. NanoSIMS Protocols 

The Cameca NanoSIMS 50L instrument (ARES Division at NASA Johnson Space Center, Houston, TX, USA) was used to conduct isotopic analyses of the cells. A focused 16 keV, ~1.5 pA Cs^+^ primary ion beam was repeatedly rastered over 20 × 20 μm^2^ fields of view divided into 256 × 256 pixels to accumulate 20 image layers. The spatial resolution was ~150 nm. The isotopes ^12^C, ^13^C, ^16^O, ^18^O, ^12^C^14^N, ^12^C^15^N, and ^32^S were simultaneously collected as negative secondary ions in electron multipliers. The mass resolving power (~7000) was sufficient to resolve any isotopic interferences, including ^12^C^1^H and ^13^C^14^N. Nitrogen isotopes are analyzed as CN. Prior to analysis, each region was pre-sputtered using a higher beam current until the count rates reached equilibrium. The pre-sputtering was also conducted to clean the sample surface of any contamination and to remove the gold-palladium coating. This ensured that measured ions derived from the cells themselves, and that ion signals were stable. The dwell time during data acquisition was 1500 µs/pixel. The depth of measurement analysis is difficult to quantify, but likely was <100 nm. The individual cells were not fully consumed during NanoSIMS analysis. The ion counts for each isotope were summed over all layers and isotopic ratios were calculated for individually defined cells (i.e., each cell was considered a region of interest [ROI]). 

The measurement precision for individual cells varied depending on the size of the cell; but on average was 5.9%, 9.8%, and 2.6% (all at 2 sigma) for ^13^C/^12^C, ^18^O/^16^O, and ^15^N/^14^N ratios, respectively. Regions of interest were identified based on NanoSIMS ^32^S and ^12^C^14^N images in which individual cells were easily recognizable and cell outlines were manually determined. Measurements of San Carlos olivine grains and kerogen isotopic standards were conducted prior to analysis of the cells to tune the instrument and ensure good reproducibility of isotopic ratios. The unlabeled cells incubated at 0 °C were measured prior to analysis of each labeled treatment. These cells served as the controls to determine the baseline isotopic compositions which should reflect the natural terrestrial abundances of the tested isotopes. The C isotopic ratios are expressed as the concentrations of ^13^C to total C; i.e., as atom percent (atom-%) of ^13^C by the formula:

[^13^C/(^13^C + ^12^C)] × 100 (atom-%) (1)

The O and N isotopic ratios are similarly expressed as the concentrations of the minor isotopes relative to the total counts of each element. Custom software written in the IDL language at NASA Johnson Space Center (JSC), Houston, TX, USA was used to process the NanoSIMS ion images and extract isotope ratios for ROIs.

### 2.6. Statistics 

**Figure 1:**
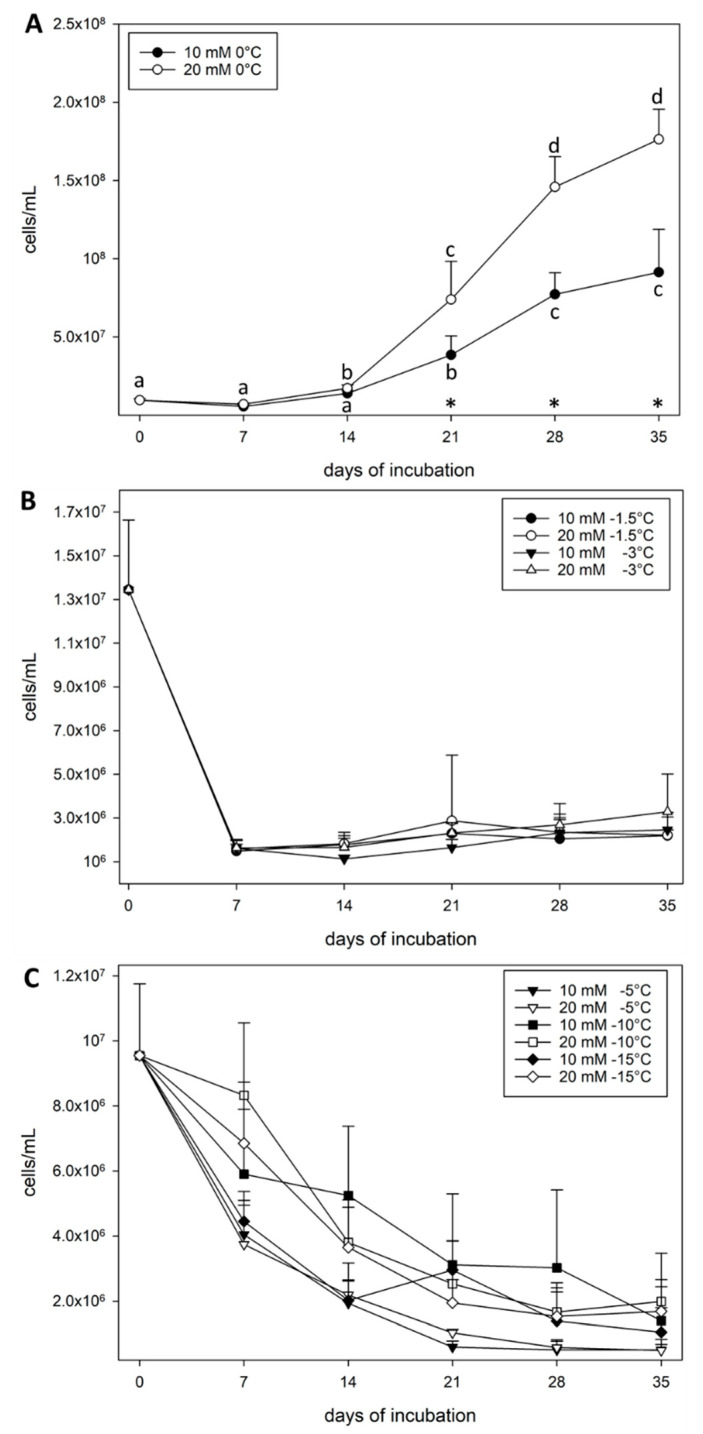
Growth curves for *Serratia liquefaciens* cells incubated at 0, −1.5, −3, −5, −10, or −15 °C in Spizizen salt medium amended with either 10 or 20 mM of glucose. Viable cell concentrations were determined via the MPN method (*n* = 6) every 7 days for 35 days. Cell concentrations are given as the number of cells/mL observed at (**A**) 0 °C, (**B**) −1.5 and −3 °C, (**C**) −5, −10, and −15 °C. Asterisks (*) at 21, 28, and 35 days in Figure 1A indicate that the 10 vs. 20 mM pairwise comparisons were significantly different (*p* ≤ 0.05; *n* = 6). Letters indicate significant differences for days for each glucose concentration tested separately. Error bars are standard errors of the means.

All data were analyzed with the Statistical Analysis System (SAS; v9.4; SAS Institute, Inc. Cary, NC, USA) and plotted with the program SigmaPlot v14.0 (Systat Software, Inc., San Jose, CA, USA). Following a Box-Cox transformation (i.e., λ = −0.25 power transformation) to achieve homogeneity of variances, growth data were analyzed using linear mixed model methodology as implemented in SAS PROC GLIMMIX using glucose concentration, day, and temperature as fixed effects. Experimental repetitions, which were not of primary interest, were treated as random blocking factors. Least-squares interactive means were calculated and back-transformed to the data scale. Standard error estimates were back-transformed in SAS using the DELTA rule. Pairwise comparisons among groups were made with consideration to the factorial nature of the experiments. For example, and regarding Figure 1 data, rather than making all possible 64 pairwise comparisons among the 12 treatments (2 glucose levels × 6 time points) we first compared days within glucose levels to day-zero using a Dunnett’s test. This was followed up with pairwise *t*-tests, comparing glucose levels at each day, excluding Day 0. Thus, the numbers of comparisons were reduced from 64 (see above) to 15 (i.e., 2 × 5 for days within glucose level plus 5 pairwise comparisons between glucose levels at days >0). A similar approach was used for all other datasets. All means are presented as back transformed numbers in tables and figures.

NanoSIMS data were analyzed and plotted using the same software as the previous experiment. Data were analyzed using linear mixed model methodology as implemented in SAS PROC GLIMMIX with treatment as the sole fixed effect, in which treatment was a combination of time and isotope labeling. In addition to labeled material—evaluated at 6 time points—there was an unlabeled control and a UV-irradiated (dead) control. Residuals were normally distributed but with unequal variances. We used R-side modeling with the above named PROC to create homogeneous variance groups. Simple *t*-tests (equal and unequal variances) were used to compare response means.

A data management plan (DMP) was developed to provide access to all raw data generated herein. All raw data are posted in the Supplementary Data linked to this paper. Furthermore, data will be posted at the University of Florida Institutional Repository (UFIR) at the website https://ufdc.ufl.edu/ir (accessed on 29 April 2021) within 90 days of the publication of this study. Search for Andrew Schuerger and then the title of the paper. 

## 3. Results

**Growth experiments of *S. liquefaciens* at 0 °C and sub-zero temperatures**. *Serratia liquefaciens* cells were incubated at 0, −1.5, −3, −5, −10, and −15 °C in either 10- or 20-mM glucose as the sole carbon source (Figure 1). After 35 days, growth was observed in cultures only for samples incubated at 0 °C (Figure 1A)—in which growth curves followed typical sigmoidal patterns with relatively long lag phases of about 14 days, followed by exponential growth for 14–21 days. However, plateaus for 10- and 20-mM glucose-incubated cells were not reached after 35 days, indicating slow growth rates at 0 °C, and thus, the nutrients in the media were not yet exhausted. 

Incubation at −1.5 and −3 °C for 35 days (Figure 1B) indicated that after an initial small drop in cell numbers, cell densities remained relatively stable over 35 days, but eventually showed positive growth after incubating cultures for a total of 70 days (Table 1). For treatments incubated at −1.5 °C and −3 °C, the cell densities increased from an average of 2 × 10^6^ cells/mL to between 3 × 10^7^ and 9 × 10^7^ cells/mL after 70 days (Figure 1B, Table 1). Summarizing between 7 and 35 days, no significant differences were observed between the temperature and glucose treatments. Data for day 70 were significantly higher compared to the starting concentrations indicating growth at −1.5 and −3 °C (*p* ≤ 0.05; *n* = 6). 

In contrast, cell densities in cultures incubated at −5, −10, and −15 °C (Figure 1C) decreased by at least ½ order of magnitude over 35 days. The data at and below −5 °C indicated that by 35 days the cells had decreased by approximately 1 order of magnitude. This growth experiment was repeated and incubated for a total of 70 days. Cell densities decreased between 1 and 2 logs at −5, −10, and −15 °C after day 70 (Table 1). All treatments significantly decreased approximately 1 order of magnitude over time (statistically significant lettering are not shown for clarity) (*p* ≤ 0.05; *n* = 6).

In addition, we tested whether the different glucose concentrations added to the medium had an effect on the growth of *S. liquefaciens*. It was shown in an earlier study [21] that *S. liquefaciens* can grow on glucose as the sole carbon source at low temperatures and low pressure. Incubation with either 10 mM or 20 mM glucose for all temperatures demonstrated that both concentrations promoted growth at 0 °C after 14 days, but that 20 mM glucose exhibited a near doubling of the cell densities per mL after 35 days compared to the 10 mM glucose (Figure 1A). For the 10 mM glucose treatments the cell numbers were not significantly different at 14 days but showed a significant increase up to 35 days (*p* ≤ 0.05). Starting at Day 21, a significant difference was observed between the growth at 10 mM or 20 mM with higher cell numbers reported for samples incubated at 20 mM glucose. In addition, both 10- and 20-mM incubations at −1.5 and −3 °C revealed a significant increase between Day 35 and Day 70 (*p* ≤ 0.05; Figure 1B, Table 1). All treatments for 10 mM and 20 mM glucose at temperatures below −5 °C were significantly different between 0 and 35 days and suggested that cells were dying (Figure 1C, Table 1). Based on the observed significantly higher growth rates and lower death rates when cells were grown at 20 mM glucose, the higher glucose concentration was used for the subsequent NanoSIMS analyses. 

**NanoSIMS imaging of *S. liquefaciens* at 0 °C and sub-zero temperatures**. NanoSIMS was used to verify the temperature extremes for cellular growth, and to determine whether metabolic activity occurred in cells that had very low or no growth over time. Individual cells in the NanoSIMS images (total of 240 cells; Table 2 presents the number of cells analyzed per treatment) were manually outlined as individual ROIs, the atom-% of ^13^C, ^15^N, and ^18^O calculated according to Equation (1), and then data for each treatment compared with the control values in unlabeled cells. 

**Figure 2:**
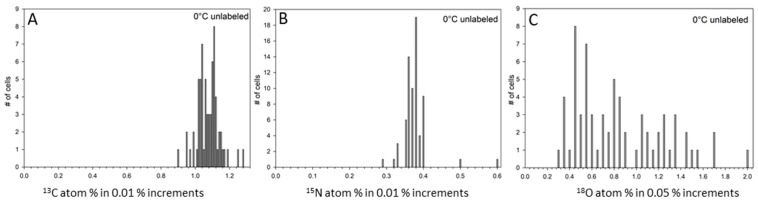
Distribution of measured ^13^C, ^15^N, and ^18^O assimilations (atom-%) for the unlabeled controls for single *Serratia liquefaciens* cells incubated at 0 °C without added labels obtained by NanoSIMS analyses. Single cell data were collected for 67 individual cells for (**A**) ^12^C and ^13^C measurements; (**B**) ^12^C^14^N and ^12^C^15^N measurements, and (**C**) ^16^O and ^18^O measurements. Data are plotted as different percentage (atom-%) increments.

**Figure 3:**
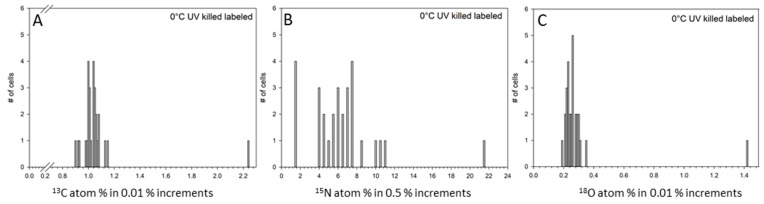
Distributions of measured ^13^C, ^15^N, and ^18^O assimilations for single UV-killed cells of *Serratia liquefaciens* incubated at 0 °C with added labels (i.e., ^13^C-glucose, ^15^N-ammonium sulfate, and H_2_^18^O) obtained by NanoSIMS analyses. These data serve as controls to infer the inactive accumulation of the stable isotope labels. Single cell data (*n* = 33) were collected for (**A**) ^13^C-labeled cells; (**B**) ^15^N-labeled cells, and (**C**) ^18^O-labeled cells for 33 individual cells. Data are plotted as different atom percentage (atom-%) increments.

In order to confirm the enrichment of ^13^C, ^15^N, and ^18^O in any given cell, a significant increase above the unlabeled control had to be detected (Figure 2; Supplemental Figure S2). Table 2 summarizes the atom-% measured for each sample and label. A second control included UV-killed cells of *S. liquefaciens* incubated for 35 days in labeled media to determine the effects of sample preparation, percentage of labels sticking to dead cells, and the inactive passive influx of labels into cells (Table 2; Figure 3; Supplemental Figure S3). Based on the results obtained from the UV-killed cells, there was no significant adsorption and uptake of ^13^C (1.04%) and ^18^O (0.63%), respectively (Table 2). However, for the N-isotopic measurements, we observed significant increases in the ^15^N labels in UV-killed cells (17.49% versus 0.38% in unlabeled cells; Table 2). Based on these observations, increased ^13^C and ^18^O label incorporation can be attributed to metabolic activity for *S. liquefaciens* cells; but for ^15^N data, only fractions significantly different from the UV-killed cells would indicate active assimilation. 

**Figure 4:**
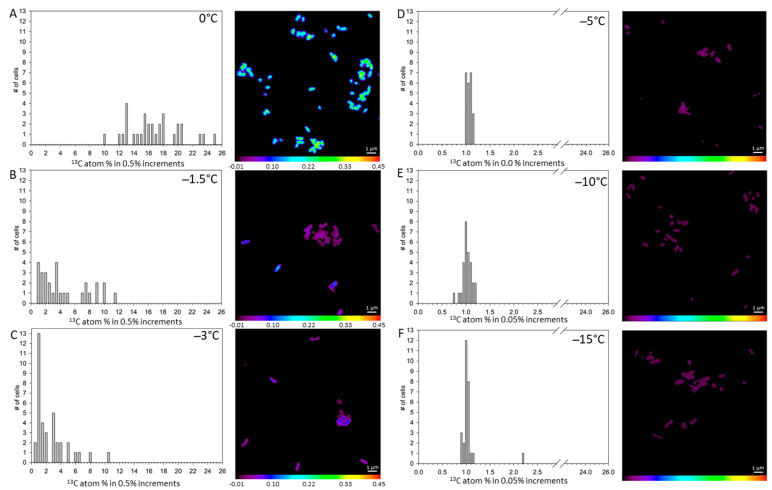
Distributions of measured ^13^C accumulations for single *Serratia liquefaciens* cells incubated at 0 °C and sub-zero conditions with added isotopic labels (^13^C-glucose, ^15^N-ammonium sulfate, and H_2_^18^O) obtained by NanoSIMS analyses. Data are plotted as different atom percentage (atom-%) increments. Single cell data and corresponding isotope ratio images for: (**A**) cells incubated at 0 °C (*n* = 31), (**B**) cells incubated at −1.5 °C (*n* = 29), (**C**) cells incubated at −3 °C (*n* = 37), (**D**) cells incubated at −5 °C, (*n* = 23), (**E**) cells incubated at −10 °C (*n* = 28), and (**F**) cells incubated at −15 °C (*n* = 28). Mean ^13^C accumulation values for each treatment are given in Table 2.

**^13^C enrichment in *S. liquefaciens* at 0 °C and sub-zero temperatures.** The distributions and levels of ^13^C atom-% in individual cells incubated between 0 and −15 °C are displayed as bar plots with their corresponding isotopic ratio images in Figure 4. Incubation of *S. liquefaciens* cells at 0 °C in ^13^C-glucose labelled growth media dramatically increased the cell concentrations of ^13^C ions (Figure 4A), confirming previous studies [19,20,21] that showed active metabolism and growth for *S. liquefaciens* under simulated Mars low-PTA conditions. The mean ^13^C atom-% calculated for samples enriched at 0 °C was 16.93 ± 0.635 atom-% (Table 2), suggesting that supplemented ^13^C-glucose in the cells were converted to labeled ^13^C macromolecules. However, as the temperature was lowered, the enrichment of ^13^C continuously decreased (Figure 4; Table 2). The determined ^13^C assimilation of *S. liquefaciens* was approximately 75 atom-% lower by the slight reduction in temperature from 0 to −1.5 °C. The ratio decreased even further when cells were incubated at −1.5 °C and −3 °C, revealing a decrease in metabolic activity with decreasing temperatures. The observed ^13^C atom-% values at 0, −1.5, and −3 °C were significantly higher than the unlabeled controls. Conversely, cells incubated at −5, −10 and −15 °C had ^13^C atom-% values that were not significantly different from the unlabeled cells incubated at 0 °C, indicating no active uptake and incorporation. Results suggest that metabolic activity was greatly reduced at −1.5 and −3 °C, halted at −5 °C, and shut-down completely at −10 and −15 °C (Table 2). 

**Figure 5:**
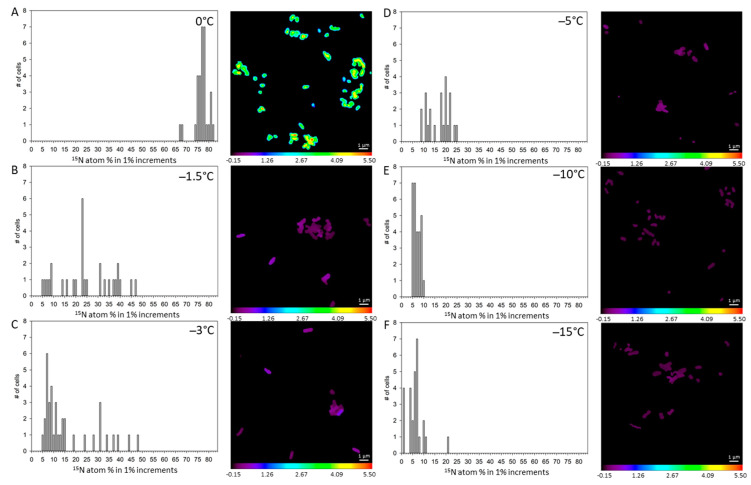
Distributions of the measured ^12^C^15^N accumulations for single *Serratia liquefaciens* cells incubated at 0 °C and sub-zero conditions with added labels (^13^C-glucose, ^15^N-ammonium sulfate, and H_2_^18^O) obtained by NanoSIMS analyses. Data are plotted with different atom percent (atom-%) increments. Single cell data and corresponding isotope ratio images for: (**A**) cells incubated at 0 °C (*n* = 31), (**B**) cells incubated at −1.5 °C (*n* = 29), (**C**) cells incubated at −3 °C (*n* = 37), (**D**) cells incubated at −5 °C, (*n* = 23), (**E**) cells incubated at −10 °C (*n* = 28), and (**F**) cells incubated at −15 °C (*n* = 28). Mean ^12^C^15^N accumulation values for each treatment are given in Table 2.

**^15^N enrichment in *S. liquefaciens* at 0 °C and sub-zero temperatures**. The highest levels of the ^15^NH_4_^+^ accumulation (77.24 ± 0.674 atom-%; Figure 5 and Table 2) incorporated into biomass were detected when *S. liquefaciens* cells were grown at 0 °C. Furthermore, ^15^N enrichment fractions decreased at sub-zero temperatures relative to 0 °C until −3 °C, consistent with the trend observed for ^13^C. Whereas the ^13^C atom-% values measured in samples incubated at −5, −10, and −15 °C (1.08, 1.05, and 1.07 atom-%, respectively) were very close to those observed in the unlabeled control samples (1.08 atom-%), the ^15^N atom-% values at these temperatures ranged from approximately 17 to 6 atom-%, significantly higher than the unlabeled control (0.38 atom-%; Table 2). However, the UV-killed *S. liquefaciens* cells exhibited 17 atom-% of ^15^N, indicating a passive enrichment by ^15^N. The data revealed that samples incubated at −3 °C and −5 °C were not significantly different from the UV-killed cells. Surprisingly, the atom-% of ^15^N in cultures incubated at −10 and −15 °C revealed ^15^N accumulations close to 7 atom-%, which were significantly lower than the values observed for the UV-killed cells but significantly higher than the values in the unlabeled cells. For cultures incubated at −1.5, −3, and −5 °C, a broader range of enrichments were observed compared to cells grown at 0 °C, −10 °C or −15 °C (Figure 5). 

**Figure 6:**
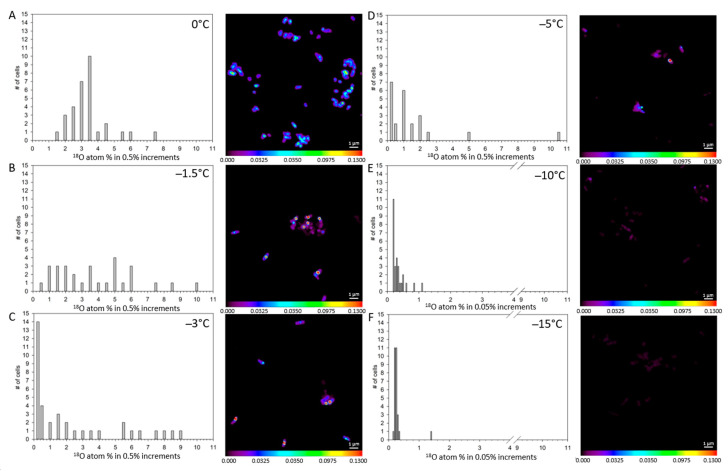
Distributions of the measured ^18^O accumulations for single *Serratia liquefaciens* cells incubated at 0 °C and sub-zero conditions with added labels (^13^C-glucose, ^15^N-ammonium sulfate, and H_2_^18^O) obtained by NanoSIMS analyses. Data are plotted with different atom percent (atom-%) increments. Single cell data and corresponding isotope ratio images for: (**A**) cells incubated at 0 °C (*n* = 31), (**B**) cells incubated at −1.5 °C (*n* = 29), (**C**) cells incubated at −3 °C (*n* = 37), (**D**) cells incubated at −5 °C, (*n* = 23), (**E**) cells incubated at −10 °C (*n* = 28), and (**F**) cells incubated at −15 °C (*n* = 28). Mean ^18^O accumulation values for each treatment are given in Table 2.

**^18^O enrichment in *S. liquefaciens* at 0 °C and sub-zero temperatures**. The distribution and level of ^18^O accumulation for individual cells are shown in Figure 6. As reported for the ^13^C and ^15^N isotopes, ^18^O enrichment followed the general trend of decreased accumulation at lower temperatures (Table 2). The values measured for the unlabeled controls were significantly different than for the UV-killed controls but were significantly higher compared to −10 °C or −15 °C exposed cells. Incubation at 0 °C and −1.5 °C led to significant uptake of ^18^O within the cells (3.64 atom-% at 0 °C and 4.06 atom-% at −1.5 °C versus 0.87 atom-% for unlabeled cells). In addition, he NanoSIMS results revealed significant ^18^O assimilation into the cell down to −3 °C. While the ^18^O atom-% values were highest for 0 and −1.5 °C, assimilation observed for −3 and −5 °C were significantly lower compared to the former but significantly higher than treatments incubated at −10 or −15 °C (Figure 6). The NanoSIMS data showed no ^18^O enrichment for cells at −10 and −15 °C; compared to the unlabeled control in fact, the values reported were lower (Figure 6; Table 2).

## 4. Discussion

Astrobiological model organisms are frequently used as proxies to study how to search for life on Mars. The basis of this assumption is that if microbial life can persist on Earth in extreme conditions that mimic environmental and geochemical conditions on Mars, then it is at least plausible that an extant microbial community might persist under similar conditions on Mars. We have begun exploring the lower limits of temperature and pressure on the metabolism and growth of microorganisms plausibly recovered from Mars spacecraft. Recently, *Serratia liquefaciens*—a mesophilic bacterium—has been characterized as a hypopiezotolerant spacecraft bacterium capable of metabolism and growth under simulated low-PTA Mars conditions of 7 hPa, 0 °C, and a CO_2_-enriched anoxic atmosphere [19,21]. Of the many microorganisms tested, only 30 species in 10 genera are currently described as hypopiezotolerant bacteria; no fungi or archaea have to-date been identified as hypopiezotolerant microorganisms. 

The bacterium *S.* *liquefaciens* ATCC 27,592 is considered a model organism for Mars simulation studies because it grows on TSA and in liquid media under low-PTA conditions [20,21,22]. However, Martian temperatures are predominately in the sub-zero range; with a global average of −61 °C [14]. Since there are no data on growth at sub-zero temperatures for *S. liquefaciens*, we investigated the potential for growth and metabolic activity down to −15 °C to assess the potential for *S. liquefaciens* to grow on Mars.

The observed growth rates for *S. liquefaciens* were significantly reduced at sub-zero temperatures compared to 0 °C, and eventually ceased at ≤−10 °C. Sub-zero temperatures likely led to exponential reductions in the reaction rates for enzymes and ultimately led to slower growth rates. Growth of *S. liquefaciens* at an Earth normal pressure of 1013 hPa was observed only for temperatures ≥−3 °C, and thus, the cultivation results herein suggest that for *S. liquefaciens*, the range for growth at temperatures below 0 °C is very narrow. However, the growth rate data given in Figure 1 do not allow us to draw conclusions about whether the cells were metabolically active below the limit for growth of −3 °C. 

NanoSIMS isotopic imaging was used to probe the limits of metabolism under sub-zero temperatures at the cellular level, compare the label incorporation between different temperatures, and determine the feasibility of using ^18^O-labeled H_2_O as an indicator for metabolic activity in environmental samples. From the comparison of labeled and unlabeled cell data, conclusions about the metabolic state of microbial cells can be drawn based on the uptake of stable isotopes. Non-dividing cells may still perform biochemical activities, protein, and ribosomal remodeling and hence may be considered metabolically active [37]. Uptake of nutrients by bacteria have been described down to −15 °C [38], and permafrost studies indicated metabolic activity approaching −40 °C [24]. To circumvent the limits of population-growth studies—which, mostly do not provide insights on cellular metabolism—NanoSIMS was used to confirm metabolic activity on a cell-by-cell basis at sub-zero temperatures. 

For NanoSIMS analysis, we incubated *S. liquefaciens* under various temperatures in Spizizen media containing the three labeled isotopes: ^13^C-glucose as the sole carbon source, (^15^NH_4_)_2_SO_4_ for nitrogen, and H_2_^18^O for oxygen incorporation. The majority of previous studies have applied different tracers such as isotopically labeled C or N substrates to investigate their incorporation into total biomass, nucleic acids, proteins or lipids in microbial cultivations or in environmental samples (e.g., [39,40,41,42,43,44]). Incubations with ^15^NH_4_^+^ substrates have been shown to allow de novo protein synthesis and growth dynamic measurements [41,45,46] and the effect of nitrogen availability on microbial community compositions [47,48]. Isotopically labeled H_2_^18^O has been mostly used for stable isotope probing, a technique in microbial ecology for tracing incorporation of labeled oxygen into DNA and RNA to characterize their synthesis as an indicator for microbial activity [49,50,51,52]. However, application of H_2_^18^O with NanoSIMS is limited [53]. 

Theoretically, the use of labeled water has certain advantages over labeled C and N compounds. First, all life is water-based and hence all microorganisms take up and incorporate labeled H_2_O, which makes it a universal bacterial substrate. Consequently, there is no bias due to a preferential selection or the inability to use another labeled substrate due to the specific metabolic capabilities of the microorganism. Second, since water is neither an energy, carbon, or nitrogen source, microbial growth rates are not affected directly [49]. Therefore, it is advantageous for characterizing environmental samples without any prior knowledge of the microbial community and metabolism.

Using NanoSIMS, the ^13^C data suggested a halt in metabolic activity at −3 °C compared to −1.5 °C as observed from the ^15^N data. However, the ^15^N data might be biased due to the relatively high accumulation of ^15^N in the UV-killed cells which might mask the real limit (see below). The ^18^O data indicated that metabolic activity ceased in *S. liquefaciens* cells down to −10 °C. Despite these variations, results of the three labeled isotopes confirm metabolic activity in the narrow range of −0 °C and −5 °C while at lower temperatures active metabolism was halted. Similar results have been reported for *Clostridium psychrophilum* incubated at sub-zero temperatures down to −15 °C [54]. However, for true psychrophiles, such as *Planococcus halocryophilus—*a permafrost microbe able to grow at −15 °C and metabolize down to −25 °C—the boundaries are more extreme [55]. The decrease of metabolic activity below −5 °C can be attributed to the fact that when bacteria are exposed to super-cooled liquid conditions (−1.5 to −5 °C) or ice formation (−10 °C to −15 °C) cell functionality and viability are influenced by several factors including an increase in water viscosity, a decrease in molecular diffusion rates, a reduction in biochemical reaction rates, and a decrease in cell membrane fluidity, intracellular ice crystal formation, and osmotic stress [54]. Furthermore, with decreasing temperature, energy required per cell division increases dramatically. These observations are supported by the growth experiments with cells dying at temperatures ≤−5 °C; which could be a consequence of plasmolysis induced by the freezing of the liquid medium.

Growth of *S. liquefaciens* was halted at −3 °C (Figure 1; Table 1) indicating that below this temperature threshold the metabolic cost of reproduction eventually became too high. Thus, the low temperatures consequently led to a total reduction of the overall cell number due to death of a fraction of the cell population, but maintenance metabolism in cells persisted. At −5 °C, metabolic activity, but not growth, was measured for *S. liquefaciens*. These data suggest that the energy is likely being funneled towards repair and maintenance metabolism of cell structures rather than reproduction and biosynthesis [56]. 

Variation in the uptake of stable isotopes within cells at the same temperature most likely reflect that cells in the sample were in different stages of growth (i.e., different metabolic states, cell division rates) at the sampling point of 35 days. Thus, these fluctuations are most likely the result of different ages of specific cells. Furthermore, the cell solutions in samples incubated at and below −1.5 °C were mixtures of mostly alive but also dead cells since a reduction in cell viability was observed (see Figure 1; Table 1). 

During growth, labels accumulate from generation to generation which then in turn leads to incrementally higher percentages of labels in the daughter cells. Therefore, heterogeneity of label uptake for individual cells within a sample may indicate different growth stages and microbial viability. The growth curves between 0 and −3 °C demonstrated growing cells that had not reached the stationary phase, whereas the cells incubated at ≤−5 °C revealed that only a fraction of the cells survived. For example, a 1-log decrease was observed for *S. liquefaciens* cells below −5 °C and suggests that the sample contained a heterogeneous cell suspension of live, inactive, and dead cells. Differences in the metabolic states hint towards heterogeneous *S. liquefaciens* populations, which have been described in previous studies [41,57]. Nevertheless, we establish here the limits of metabolic activity for *S. liquefaciens* at temperatures ≤−5 °C.

Furthermore, we observed differences in the amount of assimilation of ^15^N versus ^13^C. The uptake was generally significantly higher for ^15^N compared to ^13^C (see Table 2). These variations may be attributed to combinations of effects from sample preparations for NanoSIMS analyses, uptake and transport mechanisms and incorporation into cells, and the metabolic states of individual cells. The lower ^13^C assimilation compared to ^15^N could be the result of a potential dilution effect caused by the fixing reagent (glutaraldehyde, ^12^C_5_H_8_O_2_) which is introduced into the cell matrix forming cross-links between protein molecules. In addition, chemical fixation with glutaraldehyde leads to differential preservation of both carbon and nitrogen compounds [34]. While glutaraldehyde reacts with amine groups of proteins, purine, and pyrimidines resulting in stabilization and immobility of protein and nucleic acids, small soluble molecules such as glucose—or other soluble molecules poor in amino groups—are not fixed and may be lost during sample preparation. Loss of label during the fixation process can also occur due to changes in the cellular membranes which may cause ions and small molecules containing C, N, and O to diffuse outward [58], and therefore would affect the ^13^C, ^15^N, and ^18^O atom percentages in cells, albeit at different rates. 

In addition, the label enrichment in cells can be the result of various mechanisms. Increased accumulation mostly hints towards increased assimilation rates into cell biomass but does not imply that the ^13^C and ^15^N concentrations measured were solely due to the actual assimilation rates. The labels could be taken up and deposited in the cells which can lead to significantly different values for C, N and O labels. In addition, molecules labelled with ^13^C and ^15^N have different pathways for uptake into cells. Glucose requires active transport for uptake into a cell while ammonium sulfate enters a cell through diffusion.

Another explanation for the significantly higher ^15^N label incorporation compared to the ^13^C assimilation might be the physiological phase of the cells; i.e., new protein synthesis may be uncoupled from growth [46]. Therefore, the higher percentages of ^15^N versus ^13^C enrichment (Figure 4 and Figure 5) might indicate that the cells performed protein synthesis rather than growth. This may lead to an increased protein turnover rate which is associated with cell maintenance [46]. Furthermore, unexpectedly high ^15^N accumulation rates were observed in the UV-killed cells which were similar at −3 and −5 °C but significantly higher than the unlabeled controls. This process hampered our efforts to determine the exact threshold for metabolic activity based on the ^15^N label data alone. 

The UV-killed samples were analyzed in order to evaluate the potential and amount of abiotic assimilation. The ^15^N result is contrary to the observation from Cliff et al. [47] in which UV-killed cells of *Pseudomonas fluorescens* were not significantly different from the natural background of control cells, hence indicating no abiotic assimilation. The reason for the high ^15^N accumulation in the UV-killed *S. liquefaciens* cells can only be speculated upon at this point. It is possible that the dead *S. liquefaciens* cells accumulated ^15^N labels as a result of passive diffusion, or adsorption of ^15^N to the exteriors of the cells with ammonium sticking to proteins and cell wall constituents. This may result in a baseline accumulation of 17% which was observed for the UV-killed cells and for the samples incubated at −3 or −5 °C. For these samples, the medium remained liquid during the incubation period. However, the medium was partially frozen at −10 and −15 °C. The lower abiotic assimilation rate in the partially frozen samples (i.e., half of the threshold determined for the UV-killed cells) may indicate significantly reduced diffusion rates for ammonium sulfate in super cooled and frozen media, or that the labeled ammonium sulfate was trapped in the frozen medium. 

Compared to ^13^C and ^15^N, the measured incorporation of ^18^O was lower. This observation could be due to a different turnover rate, stability of the molecules, and different mechanisms of incorporation of C, N and O. Furthermore, despite the fact that the ^16^O from water was fully replaced by ^18^O (97%), other medium components such as glucose, ammonium sulfate, etc. contain ^16^O which may have increased the ^16^O fraction available in the medium. Consequently, the overall percentage of ^18^O was significantly below 97%, which then in turn led to lower accumulation of ^18^O because ^16^O likely competed with ^18^O. Labeled ^18^O is mainly incorporated into DNA and RNA. For RNA it has been shown that the branch oxygen atoms of the phosphodiester linkage were rapidly labeled with ^18^O. However, not every oxygen atom in RNA is derived from H_2_O, and therefore studies found that RNA is not fully labeled with ^18^O [50]. For example, C-3 and C-5 bridge oxygen atoms originate from glucose [59]. Labeled ^18^O from water can be incorporated into phosphodiester backbones of DNA by the exchange of oxygen atoms between adenosine triphosphate (ATP), inorganic phosphate, and water. It has been suggested that nucleotides do not contain ^18^O from water if an oxygen-containing carbon source is available [51]. The results indicate that even at −5 °C, a fraction of the *S. liquefaciens* cells possessed the physiological potential for DNA and RNA metabolism. These data are in line with the growth experiments that indicate a fraction of the cells appeared to die over time (Figure 1), whereas the surviving cells had the potential capability to repair RNA and DNA damage. 

Despite the low incorporation levels, labeled H_2_^18^O can be applied in future NanoSIMS experiments to track ^18^O uptake and may provide information on the cell’s capability to counteract chromosomal damage induced by adverse environmental conditions such as UV radiation, ionizing radiation, the presence of reactive oxygen species and desiccation, or by amino acid racemization and entropic degradation. Furthermore, RNA and DNA maintenance costs are relatively small compared to protein maintenance which is thought to be the dominant energy requiring process [60].

Summarizing, in this study we have shown that the application of NanoSIMS provided a direct link between the applied environmental parameters and metabolic function on a cell-by-cell basis in low-temperature environments on Earth and has potential for Martian simulation studies. 

**Implications for Martian habitability.** There is significant interest on whether spacecraft microorganisms can establish themselves in the Martian terrain because such a process would have significant implications for the potential habitability of Mars and the risks to forward contamination of landing sites. In order for microorganisms to thrive on Mars, their ability to survive, metabolize, replicate, and adapt to low temperatures are prerequisites. On Mars, the temperatures can increase above 0 °C in the low- to mid-latitudes reaching approximately 20 °C at the equator in summer; but at the poles, the minimum temperature is −153 °C and water exists only as ice or vapor [14]. There is experimental evidence that microorganisms cannot only survive but also grow in glacial ice on Earth. Icy environments such a glacial ice or permafrost possess liquid veins [61], impurities such as clay grains [62], and within ice crystal lattice [63] can be used as microhabitats to provide the essential elements for growth. However, on Mars, pure liquid water can be maintained only within a very narrow range close to the triple point of water (i.e., 0.01 °C at 6.1 hPa [14]). The stability of water decreases rapidly and water ice sublimes with increasing temperatures. 

Previous experiments with a diversity of bacteria incubated under simulated Martian low-PTA conditions [20] demonstrated that the majority of the bacteria tested were unable to grow (i.e., evaluated by visually detecting colonies of hypopiezotolerant species). However, the data did not provide insights on whether the cells were metabolically active or replicating at extremely slow rates, and thus, not visually detected. Tracking isotopically labeled substrates into microbial biomass improves our understanding of the role that temperature plays on the growth and metabolism of *S. liquefaciens* at sub-zero conditions. For example, *S. liquefaciens* has only a narrow range for growth and metabolic activity down to −5 °C. Further, because the global average temperature on Mars is −61 °C, it seems unlikely that *S. liquefaciens* would grow on the surface of Mars even if mild salt brines, and abundant organics and micronutrients were present. Lastly, despite the indirect evidence of transient liquid water on Mars [14], the combination of low water activity, temperature, solar UV irradiation, and other inhibitory or biocidal environmental factors [19] would likely prohibit the growth of hypopiezotolerant bacteria like *S. liquefaciens* on Mars. Additional work is required to further characterize the low-pressure, low-temperature, and high-salt conditions similar to Mars on the survival, growth, and adaptation of *S. liquefaciens* and other hypopiezotolerant bacteria. 

## Figures and Tables

**Table 1:** Growth of *Serratia liquefaciens* cells incubated at 0, −1.5, −3, −5, −10, or −15 °C in Spizizen Scheme 10 or 20 mM of glucose for 70 days. Data were obtained from MPN assays (*n* = 6) and are expressed as mean viable cells per milliliter. Errors are expressed as standard errors of the means.

Viable Cells per mL	T_0_	T_70_				
		−1.5 °C	−3 °C	−5 °C	−10 °C	−15 °C
10 mM	7.2 × 10^7^	6.5 × 10^7^	3.2 × 10^7^	5.8 × 10^4^	1.2 × 10^5^	1.1 × 10^5^
	±1.7 × 10^7^	±3.0 × 10^7^	±1.7 × 10^7^	±1.7 × 10^4^	±1.8 × 10^4^	±4.2 × 10^4^
20 mM	7.2 × 10^7^	9.4 × 10^7^	4.3 × 10^7^	7.2 × 10^4^	2.5 × 10^5^	1.5 × 10^5^
	±1.7 × 10^7^	±4.0 × 10^7^	±1.7 × 10^7^	±4.4 × 10^2^	±1.7 × 10^3^	±1.7 × 10^3^

**Table 2:** Summary of the NanoSIMS analyses. Accumulations of ^13^C, ^15^N and ^18^O isotopes are reported as atom-percentages (atom-%) *Serratia liquefaciens* cells incubated at 0 °C and sub-zero temperatures for 35 d. Ultraviolet (UV) killed cells were measured to determine the abiotic assimilation of the isotopes. The number of ROIs (i.e., individual cells) analyzed per sample is indicated in parentheses. Fractional values were analyzed as described in the text (*p* ≤ 0.05; n varied between 23 and 67 cells per treatment). Letters in columns (tested separately) indicate significant differences among treatments. The error values are standard errors of the means.

Treatment	^13^C atom-%	Std Error	^15^N atom-%	Std Error	^18^O atom-%	Std Error
0 °C unlabeled (*n* = 67)	1.08 ^d^	0.008	0.38 ^e^	0.005	0.87 ^c^	0.050
0 °C UV-killed (*n* = 33)	1.04 ^e^	0.0034	17.49 ^c^	0.303	0.63 ^d^	0.013
0 °C labeled (*n* = 31)	16.93 ^a^	0.635	77.28 ^a^	0.674	3.63 ^a^	0.217
−1.5 °C labeled (*n* = 29)	4.70 ^b^	0.601	25.08 ^b^	2.429	4.06 ^a^	0.424
−3 °C labeled (*n* = 37)	2.83 ^c^	0.373	16.97 ^c^	1.904	2.47 ^b^	0.472
−5 °C labeled (*n* = 23)	1.08 ^d^	0.010	17.66 ^c^	0.908	1.75 ^bc^	0.535
−10 °C labeled (*n* = 28)	1.05 ^de^	0.018	7.51 ^d^	0.358	0.37 ^e^	0.041
−15 °C labeled (*n* = 28)	1.07 ^de^	0.045	6.75 ^d^	0.746	0.30 ^e^	0.041

## Supplementary Materials

The following are available online at https://www.mdpi.com/article/10.3390/life11050459/s1, Figure S1*:* examples of scanning electron microscopy (SEM) images of *Serratia liquefaciens* incubated in labeled Spizizen medium for 35 days at 0 °C (A) or −5 °C (B). Figure S2: NanoSIMS isotopic ratio images of *S. liquefaciens* cells incubated for 35 days in 0 °C in unlabeled Spizizen medium with (A) ^13^C/^12^C, (B) ^15^N/^14^N, and (C) ^18^O/^16^O. Figure S3: NanoSIMS isotope ratio images of UV-killed *S. liquefaciens* cells incubated for 35 days in 0 °C in labeled Spizizen medium with (A) ^13^C/^12^C, (B) ^15^N/^14^N, and (C) ^18^O/^16^O.
