# Supplementary material for: Use of NanoSIMS to Identify the Lower Limits of Metabolic Activity and Growth by Serratia liquefaciens Exposed to Sub-Zero Temperatures
Source: Life (Basel). 2021 May 20;11(5):459. doi: 10.3390/life11050459 (PMC8161314; doi:10.3390/life11050459)
Supplement: Supplementary file 1 [file life-11-00459-s001.zip › life-1222721-supplementary.pdf]

life

MDPI

Article

Use of NanoSIMS to Identify the Lower Limits of Metabolic Activity and Growth by *Serratia liquefaciens* Exposed to Sub-zero Temperatures

Petra Schwendner [1,*], Ann N. Nguyen [2,†] and Andrew C. Schuerger [1]

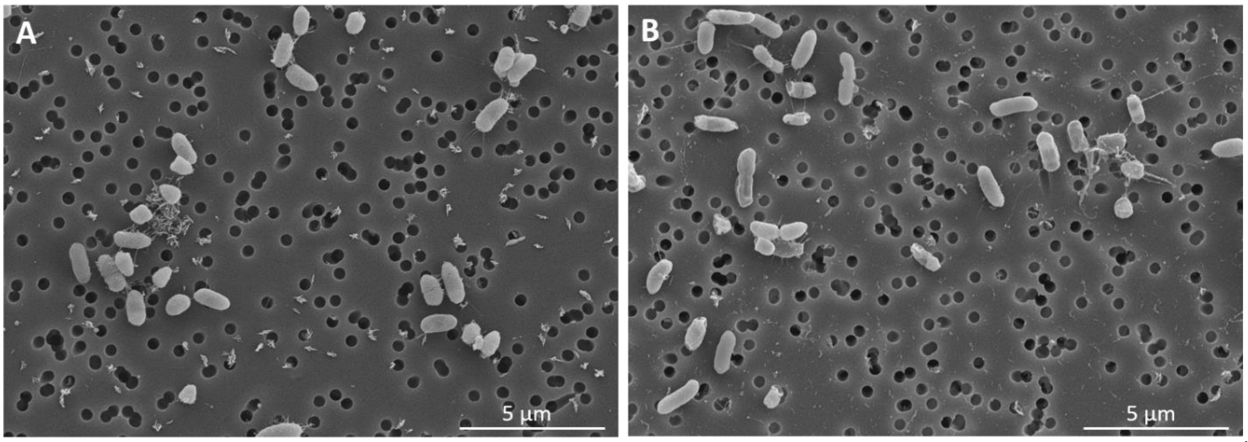

Figure S1. Examples of scanning electron microscopy (SEM) images of *Serratia liquefaciens* incubated in labeled Spizizen medium for 35 days at 0°C (**A**) and –5°C (**B**).

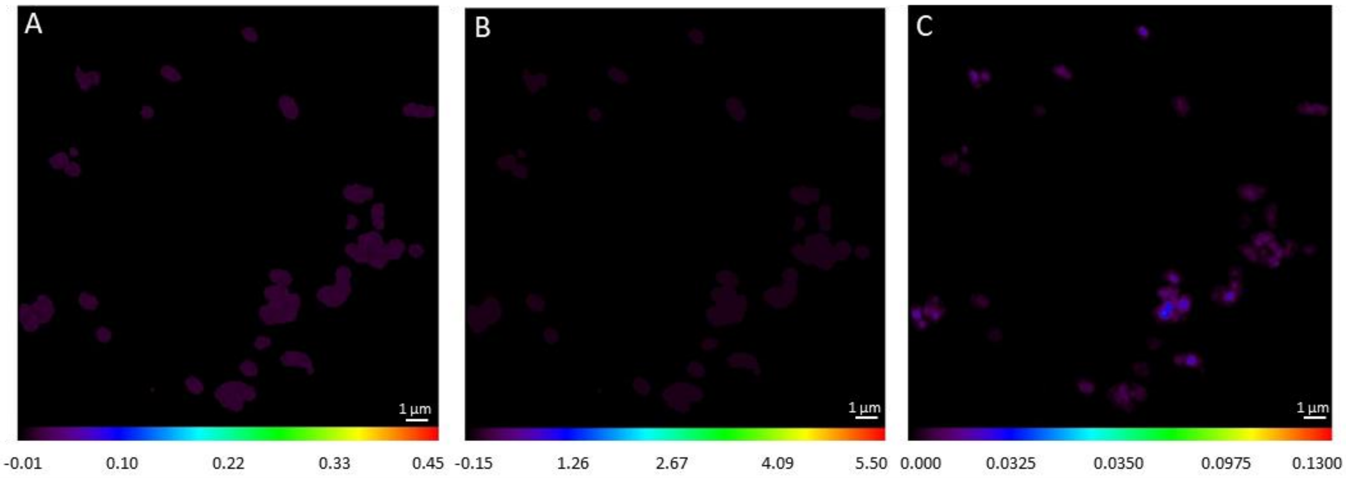

Figure S2. NanoSIMS isotopic ratio images of *Serratia liquefaciens* cells incubated for 35 days in 0°C in unlabeled Spizizen medium. (**A**) $^{13}C/^{12}C$, (**B**) $^{15}N/^{14}N$, and (**C**) $^{18}O/^{16}O$.

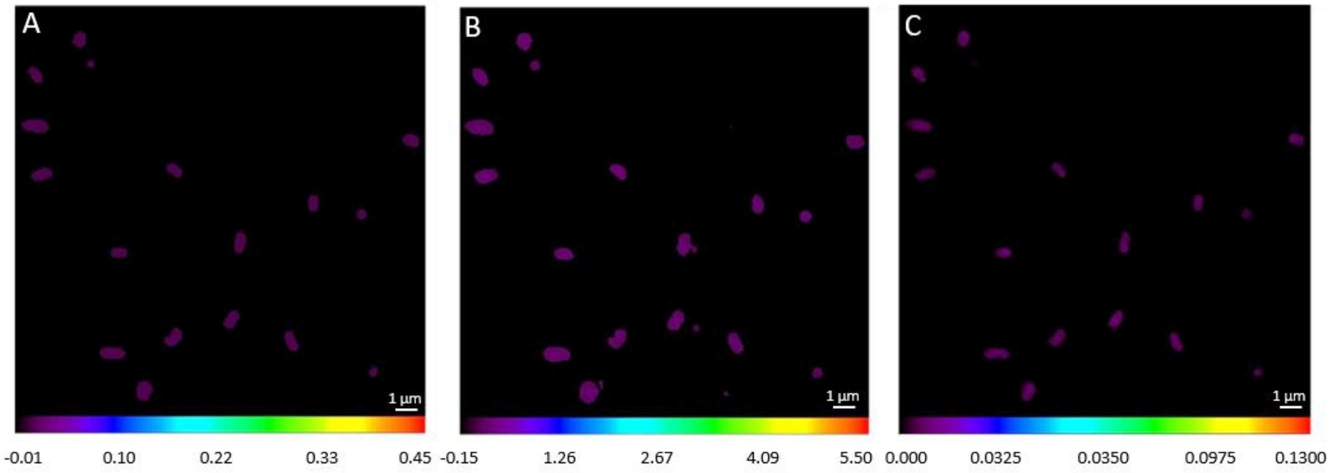

Figure S3: NanoSIMS isotope ratio images of UV-killed *S. liquefaciens* cells incubated for 35 days in 0 °C in labeled Spizizen medium with (A) $^{13}C/^{12}C$, (B) $^{15}N/^{14}N$, and (C) $^{18}O/^{16}O$.
